# Hantavirus Emergence in a Changing World: Virology, Pathogenesis, Surveillance, and One Health Preparedness

**DOI:** 10.3390/microorganisms14061326

**Published:** 2026-06-13

**Authors:** Maria E. Ramos-Nino, Nicolette Tiffanie Chiem, Prakash V. A. K. Ramdass

**Affiliations:** 1 Department of Microbiology, Immunology, and Pharmacology, School of Medicine, St. George’s University, St. George P.O. Box 7, Grenada; mramosni@sgu.edu; 2 Department of Public Health and Preventive Medicine, School of Medicine, St. George’s University, St. George P.O. Box 7, Grenada; nchiem@sgu.edu

**Keywords:** hantavirus, HFRS, HCPS, virology, disease surveillance, emerging infectious diseases, zoonoses, rodent-borne pathogens, climate change, pathogenesis, One Health, Caribbean

## Abstract

Hantaviruses are emerging rodent-borne pathogens that pose increasing global public health concerns due to their association with hemorrhagic fever with renal syndrome (HFRS) and hantavirus cardiopulmonary syndrome (HCPS), both of which can result in substantial morbidity and mortality. Environmental change, climate variability, urbanization, and land-use transformation are increasingly recognized as critical drivers of hantavirus emergence and transmission. This review summarizes current evidence regarding hantavirus virology, epidemiology, pathogenesis, clinical manifestations, diagnostics, surveillance systems, prevention strategies, and One Health preparedness approaches. Emphasis is placed on the influence of climate change and ecological disruption on rodent reservoir dynamics and spillover risk, as well as major surveillance and diagnostic gaps in tropical and Caribbean regions where hantavirus circulation may be underrecognized. Advances in molecular diagnostics, genomic surveillance, vaccine development, monoclonal antibody therapies, and climate-based early warning systems are also discussed. Existing evidence highlights the importance of integrated One Health surveillance systems that combine human, animal, and environmental monitoring to improve early detection and outbreak preparedness. Strengthening laboratory capacity, ecological surveillance, regional collaboration, and public health infrastructure will be essential for reducing the global burden of hantavirus infections and improving preparedness for future zoonotic disease threats.

## 1. Introduction

The emergence and re-emergence of zoonotic infectious diseases continue to threaten global health, specifically in the setting of climate change, ecological disruption, urbanization, and increasing human mobility [1]. Among these pathogens, hantaviruses are an important group of rodent-borne viruses belonging to the family *Hantaviridae* that can cause severe and potentially fatal disease in humans [2]. Since the recognition of hantavirus cardiopulmonary syndrome (HCPS) in the southwestern United States in 1993, growing evidence of hantavirus circulation across Asia, Europe, and the Americas has increased concern regarding their epidemic potential and public health impact [3]. Hemorrhagic fever with renal syndrome (HFRS) accounts for thousands of hospitalizations annually, notably in Asia [4], while HCPS remains associated with high mortality rates that range from 20 to 50% in the Americas [5,6].

Hantaviruses are maintained in nature through persistent infection of specific rodent reservoir hosts, which shed virus in urine, feces, and saliva without developing significant disease [7]. Human infection most commonly occurs through inhalation of aerosolized viral particles from contaminated rodent excreta, although rodent bites and rare cases of person-to-person transmission, primarily involving Andes virus, have also been reported [8]. Consequently, hantavirus epidemiology is intricately linked to rodent ecology, environmental conditions, and patterns of human exposure [9].

Environmental and anthropogenic changes, such as climate variability, deforestation, urbanization, agricultural expansion, and land-use change, are increasingly influencing hantavirus transmission by altering rodent ecology and human–rodent interactions [10,11]. Climate-related events such as El Niño Southern Oscillation (ENSO) cycles and altered rainfall patterns have been linked to increased hantavirus incidence in endemic regions [12,13,14]. These concerns are especially relevant in tropical and subtropical regions, including parts of Latin America and many island nations in the Caribbean, where favorable ecological conditions and limited surveillance infrastructure may contribute to underdiagnosis [15]. This may occur because hantavirus infections can overlap clinically with other endemic febrile illnesses, such as dengue, leptospirosis, malaria, and rickettsial infections [16,17].

Furthermore, the coronavirus disease 2019 (COVID-19) pandemic highlighted the consequences of delayed pathogen detection, inadequate surveillance, and limited outbreak preparedness for emerging infectious diseases [18]. These lessons emphasize the need for integrated surveillance systems, improved diagnostic infrastructure, rapid data sharing, and coordinated public health responses [19]. In this context, a One Health approach, integrating human, animal, and environmental surveillance may enhance early detection of hantavirus circulation and strengthen preparedness, particularly in under-surveilled and resource-limited settings [20,21].

Several comprehensive reviews have catalogued global hantavirus ecology, epidemiology, and clinical syndromes, most notably the seminal work by Jonsson et al. [5]. However, these existing reviews have only briefly addressed the unique challenges and surveillance gaps specific to tropical and Caribbean settings, where diagnostic infrastructure is limited and hantavirus infections are likely underrecognized due to clinical overlap with endemic febrile illnesses [22,23]. The present review explicitly differentiates itself by focusing on the Caribbean region as an under-surveilled hotspot, systematically evaluating the environmental and ecological drivers of hantavirus transmission in tropical island ecosystems, and assessing the applicability of One Health strategies in resource-limited Caribbean contexts. This regional emphasis, largely absent from prior comprehensive reviews, represents the primary contribution of this work.

This review summarizes current evidence regarding hantavirus virology, epidemiology, pathogenesis, clinical manifestations, diagnostics, surveillance strategies, and prevention. Emphasis is placed on the environmental and ecological drivers of hantavirus emergence, surveillance and preparedness gaps in tropical and Caribbean regions, and the role of integrated One Health approaches in strengthening global preparedness and outbreak response.

## 2. Virology and Classification

### 2.1. Viral Structure and Genome Organization

Hantaviruses are enveloped, negative-sense, single-stranded RNA viruses belonging to the family *Hantaviridae*, order *Bunyavirales* [24]. The viral genome consists of three segments: small (S), medium (M), and large (L), encoding the nucleocapsid protein (N), the glycoprotein precursor (GPC, which is cleaved into Gn and Gc envelope glycoproteins), and the RNA-dependent RNA polymerase (L protein), respectively [24]. The nucleocapsid protein is highly immunogenic and serves as the primary target for serological diagnostics, while the glycoproteins mediate viral entry and are targets for neutralizing antibodies [25].

### 2.2. Old World and New World Hantaviruses

Hantaviruses are classified into Old World and New World lineages based on their geographic distribution and phylogenetic relationships [24]. Old World hantaviruses, mostly found in Asia and Europe, are associated with HFRS and include Hantaan virus (HTNV) [26], Seoul virus (SEOV) [27], Puumala virus (PUUV) [28], and Dobrava-Belgrade virus (DOBV) [29]. These viruses are carried by rodents of the family Muridae, with each virus typically associated with a specific rodent host [30].

New World hantaviruses, found in the Americas, are associated with HCPS and include Sin Nombre virus (SNV) [31], Andes virus (ANDV) [32], and numerous other species [33]. These viruses are primarily carried by rodents of the family *Cricetidae*, subfamily *Sigmodontinae* [30]. The Andes virus is notable for being the only hantavirus with documented person-to-person transmission [34]. Recent discoveries have expanded the recognized host range of hantaviruses beyond rodents to include shrews, moles, and bats. Bat-associated hantavirus-like viruses identified in several geographic regions have broadened understanding of hantavirus diversity and evolutionary history. Although these viruses have not been clearly linked to human disease, their discovery suggests that hantavirus evolution may involve a combination of long-term host associations, host-switching events, and ecological adaptation rather than strict host–virus cospeciation alone. Together with shrew- and mole-associated hantaviruses, these findings support a more complex evolutionary history than previously recognized and highlight the ecological diversity of hantavirus reservoirs [35,36,37].

**Figure 1:**
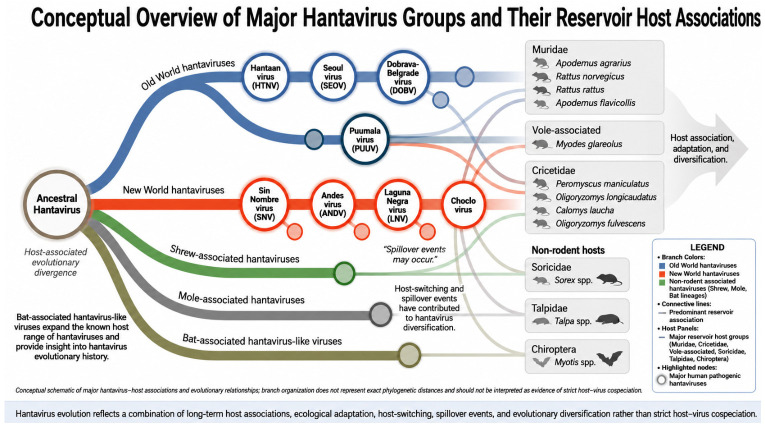
Conceptual evolutionary and reservoir host relationships of major hantaviruses. Schematic illustrating the evolutionary relationships and predominant reservoir host associations of major Old World hantaviruses (blue), including Hantaan (HTNV), Seoul (SEOV), Dobrava-Belgrade (DOBV), and Puumala (PUUV) viruses, and New World hantaviruses (red/orange), including Sin Nombre (SNV), Andes (ANDV), Laguna Negra (LNV), and Choclo viruses. Major rodent reservoirs and additional shrew-, mole-, and bat-associated hantavirus-like viruses are shown to highlight the ecological diversity of hantavirus hosts and the expanding understanding of hantavirus evolutionary history. Although many hantaviruses exhibit long-term associations with particular reservoir hosts, current evidence suggests that both host–virus co-divergence and host-switching events have contributed to hantavirus diversification. Bat-, shrew-, and mole-associated hantaviruses further support a more complex evolutionary history than previously recognized and expand the known host range of hantavirus-like viruses. The figure emphasizes the complex patterns of host association, ecological adaptation, divergence, and spillover that have shaped hantavirus evolution.

Although many hantaviruses exhibit long-term associations with particular reservoir hosts, current evidence suggests that both host–virus co-divergence and host-switching events have contributed to hantavirus diversification and geographic spread. Consequently, evolutionary relationships among hantaviruses are increasingly viewed as the result of complex interactions among host adaptation, ecological opportunity, spillover events, and viral diversification rather than strict host–virus cospeciation [38,39]. Figure 1 shows a conceptual overview of the major hantavirus groups and their reservoir host associations.

### 2.3. Host Specificity and Viral Persistence

Each hantavirus species typically exhibits a high degree of host specificity, with persistent infection in a single primary rodent reservoir species [5]. The virus establishes chronic infection in the reservoir host without causing apparent disease, facilitating long-term viral shedding in urine, feces, and saliva [40]. This host–virus co-evolution has resulted in distinct geographic distributions of hantavirus species that mirror the distributions of their rodent hosts [30]. The major hantaviruses, their reservoirs, clinical syndrome, and geographic distribution are shown in Table 1.

## 3. Global and Regional Epidemiology

### 3.1. Global Burden and Distribution

**Figure 2:**
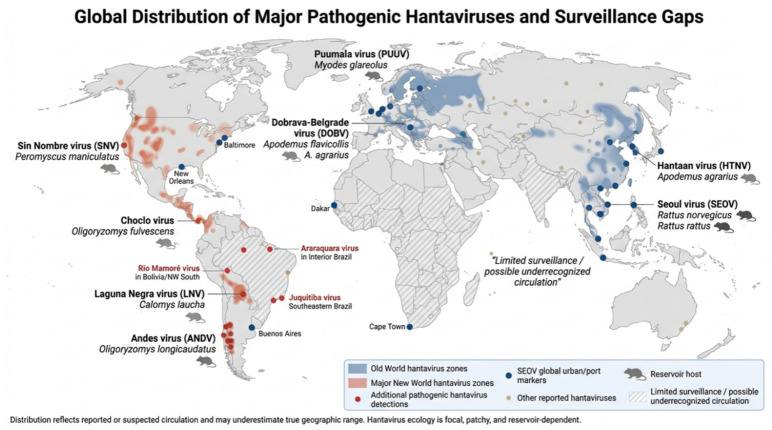
Global distribution of major pathogenic hantaviruses associated with human disease, highlighting the distinct geographic patterns of Old World hantaviruses linked to hemorrhagic fever with renal syndrome (HFRS) and New World hantaviruses associated with hantavirus cardiopulmonary syndrome (HCPS). Seoul virus (SEOV) is depicted as globally distributed due to its association with urban rats and international trade. Gray hatched regions indicate areas with limited surveillance and possible underrecognized circulation, including parts of tropical Africa, the Caribbean, tropical Latin America, and South and Southeast Asia. Distribution patterns are approximate and emphasize the focal and ecologically heterogeneous nature of hantavirus transmission.

Hantavirus infections represent a notable global public health burden, with an estimated 150,000–200,000 hospitalizations annually, predominantly due to HFRS in Asia [42]. China accounts for the majority of global HFRS cases, with tens of thousands of cases reported annually [43,44]. The Republic of Korea, Russia, and several European countries also report substantial HFRS incidence [45]. In the Americas, HCPS cases are reported from Canada to southern Argentina, with the highest incidence in rural and peri-urban areas where human-rodent contact is frequent [8]. The global distribution of major hantaviruses and associated surveillance gaps is summarized in Figure 2.

### 3.2. Hemorrhagic Fever with Renal Syndrome (HFRS) Distribution

HFRS is endemic in Asia and Europe, with the highest burden in China, where Hantaan and Seoul viruses are the predominant causative agents [46]. The disease exhibits distinct seasonal patterns, with peak incidence typically occurring in late autumn and early winter, coinciding with increased rodent activity and agricultural harvests [47,48]. Puumala virus causes a milder form of HFRS known as nephropathia epidemica in Northern and Central Europe, with thousands of cases reported annually [49,50]. Climate variables, rodent population dynamics, and land-use changes have been associated with regional variations in HFRS incidence and transmission patterns [51].

### 3.3. Hantavirus Cardiopulmonary Syndrome (HCPS) Distribution

HCPS was first recognized in the southwestern United States in 1993 during an outbreak caused by Sin Nombre virus [31]. Since then, HCPS cases have been reported throughout the Americas, with notable endemic areas in Argentina, Chile, Brazil, Paraguay, and the United States [7,52]. The disease exhibits high case fatality rates, typically ranging from 20 to 32% [53,54], with some outbreaks reporting rates exceeding 40–50% [55,56]. Environmental and climatic factors, including El Niño-associated rainfall variability and land-use change, have been linked to increased HCPS transmission risk in several endemic regions [12,13].

### 3.4. Underrepresented Regions: Tropical and Caribbean Contexts

Despite the presence of diverse rodent populations and ecological conditions favorable for hantavirus maintenance, tropical and Caribbean regions remain significantly understudied with respect to hantavirus surveillance [15,57]. Available evidence, though fragmented, indicates that hantavirus exposure occurs across several Caribbean islands and territories. The most comprehensive human data come from Barbados, where a retrospective study of febrile patients found that 15.3% of those with acute hantavirus IgM also had detectable IgG, indicating past infection [58]. Rodent surveillance in Barbados further detected orthohantavirus antibodies in 3.8% of wild *Mus musculus*, *Rattus rattus*, and *Rattus norvegicus*, although RT-PCR for viral RNA was negative, precluding species identification [59]. In Trinidad, a serosurvey of farm, abattoir, and office workers reported an IgG seroprevalence of 11.4%, representing the first documented evidence of human hantavirus exposure in the English-speaking Caribbean [60]. A single case of HCPS in an Italian traveler who visited natural reserves, rural areas, and caves in Cuba provided evidence of local hantavirus circulation, with molecular testing confirming SNV infection [61]. In Colombia’s Caribbean region (Córdoba department), a study of health center patients found a lower IgG seroprevalence of 1.0% [62]. Notably, the only documented hantavirus serosurvey including multiple Caribbean nations—Antigua, Belize, Bermuda, Dominica, Grenada, Jamaica, Montserrat, St. Kitts, St. Lucia, and St. Vincent—tested 442 healthy pregnant women and found zero seropositive samples [23]. Similarly, a study in Suriname testing patients with suspected leptospirosis also reported no hantavirus antibodies [22]. These negative findings, however, likely reflect sampling of low-risk populations (healthy pregnant women, leptospirosis suspects) rather than true absence of virus.

Collectively, these data confirm that hantavirus circulation occurs in at least four Caribbean locations (Barbados, Trinidad, Cuba, and Colombia’s Caribbean coast), yet the true regional burden remains unknown due to critical gaps. Except for the single case of the Italian traveler to Cuba, no study has successfully sequenced a Caribbean hantavirus [61]. RT-PCR was either not performed or yielded negative results, and confirmatory neutralization tests were negative. The low seroprevalence in Colombian health center patients compared to the higher rates in febrile patients in Barbados and occupational groups in Trinidad underscores the importance of targeted surveillance in high-risk populations rather than general or low-risk cohorts [58,60,62]. Furthermore, the absence of molecular characterization means that the circulating viral species—whether Seoul-like, Puumala-like, or a novel orthohantavirus—remain unidentified. Strengthening diagnostic infrastructure to include RT-PCR and virus neutralization assays, conducting systematic rodent trapping with molecular testing, and integrating hantavirus testing into routine febrile illness surveillance are essential priorities for the Caribbean region. Until these investments are made, hantavirus infections will continue to be underdiagnosed and misattributed to co-endemic illnesses such as dengue, leptospirosis, and rickettsioses [22,23]. Details of the seroprevalence of hantaviruses in the Caribbean are shown in Table 2.

## 4. Pathogenesis and Immunopathology

### 4.1. Viral Entry and Cellular Tropism

Hantaviruses enter human cells chiefly through interactions between viral glycoproteins (Gn and Gc) and cellular receptors, including integrins [63]. The primary cellular targets are vascular endothelial cells, where viral replication occurs without direct cytopathic effects [64]. This endothelial tropism is central to the pathogenesis of both HFRS and HCPS, as endothelial dysfunction leads to increased vascular permeability and the characteristic clinical manifestations of these diseases [65,66].

### 4.2. Immune Response and Immunopathology

**Figure 3:**
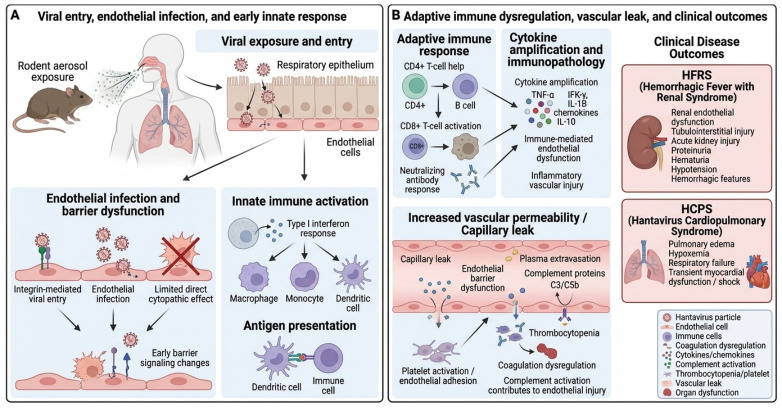
Mechanisms of hantavirus pathogenesis. (**A**) Viral entry, endothelial infection, and early innate immune responses. Hantavirus infection is typically initiated through inhalation of aerosolized rodent excreta, followed by infection of endothelial cells through integrin-mediated entry pathways. Early host responses include endothelial barrier alterations, type I interferon signaling, activation of macrophages and monocytes, and dendritic cell-mediated antigen presentation. (**B**) Adaptive immune dysregulation, vascular leak, and clinical outcomes. Adaptive immune responses involve CD4+ T-cell help, CD8+ T-cell activation, and neutralizing antibody production that contribute to viral control. In severe disease, cytokine amplification and immune-mediated endothelial dysfunction promote increased vascular permeability, complement activation, thrombocytopenia, platelet–endothelial interactions, and coagulation dysregulation, resulting in capillary leak and organ injury. These processes contribute to the development of hemorrhagic fever with renal syndrome (HFRS) and hantavirus cardiopulmonary syndrome (HCPS), with disease severity reflecting the balance between protective antiviral immunity and immune-mediated vascular injury.

The pathogenesis of hantavirus disease is largely immune-mediated rather than directly cytopathic [67]. Following infection, both innate and adaptive immune responses are activated, with the production of pro-inflammatory cytokines, chemokines, and cellular immune responses [68]. While these responses are essential for viral control, excessive or dysregulated immune activation contributes to disease severity [67]. Neutralizing antibodies targeting the viral glycoproteins play a critical role in protection and recovery [69]. Studies have demonstrated that early and robust neutralizing antibody responses are associated with better clinical outcomes [69]. Conversely, delayed or inadequate antibody responses may contribute to severe disease [70]. T cell responses, especially CD8+ T cells, are also crucial for viral clearance but may contribute to immunopathology when dysregulated [71]. Key mechanisms contributing to hantavirus pathogenesis are summarized in Figure 3.

### 4.3. Vascular Permeability and Organ Dysfunction

The hallmark of hantavirus disease is increased vascular permeability resulting from endothelial dysfunction [16]. In HFRS, this manifests as capillary leakage in the kidneys, leading to acute kidney injury, proteinuria, and hematuria [72]. The disease typically progresses through five phases: febrile, hypotensive, oliguric, diuretic, and convalescent [73]. Severe cases may develop hemorrhagic complications, shock, and multi-organ failure [74].

**Figure 4:**
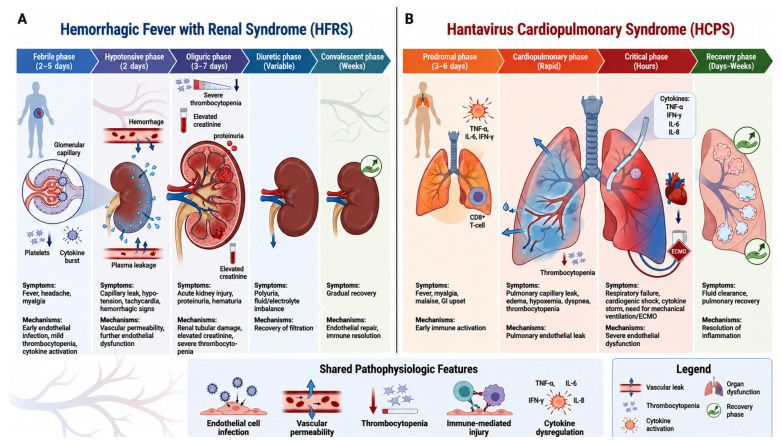
Dual-panel schematic comparing the clinical progression and major pathophysiologic features of hemorrhagic fever with renal syndrome (HFRS) (Panel (**A**)) and hantavirus cardiopulmonary syndrome (HCPS) (Panel (**B**)). Both syndromes share common pathogenic mechanisms, including endothelial cell infection, immune-mediated inflammation, thrombocytopenia, cytokine dysregulation, and increased vascular permeability leading to capillary leak and organ dysfunction. While HFRS primarily affects the kidneys and HCPS predominantly affects the lungs and cardiovascular system, both diseases result from a combination of viral infection and host immune responses that contribute to endothelial dysfunction and tissue injury.

In HCPS, vascular permeability primarily affects the pulmonary vasculature, leading to non-cardiogenic pulmonary edema, respiratory failure, and cardiogenic shock [75]. The rapid progression from initial symptoms to respiratory failure and shock is characteristic of HCPS and contributes to its high mortality rate [76]. Myocardial dysfunction, likely mediated by immune mechanisms and cytokine storm, further complicates the clinical picture [77]. The clinical progression and major pathophysiologic differences between HFRS and HCPS are illustrated in Figure 4.

### 4.4. Host Genetic Factors

Host genetic factors significantly influence susceptibility and severity of hantavirus disease, extending beyond previously recognized human leukocyte antigen (HLA) polymorphisms [78]. Specific HLA alleles are associated with either risk (e.g., HLA-B8, DRB1*0301 for Puumala virus; HLA-B*46, DRB1*09 for Hantaan virus) or protection (e.g., HLA-DRB1*12) across Old World hantaviruses causing HFRS, with emerging but less defined associations for New World viruses causing HCPS [79,80,81]. Critically, a non-HLA polymorphism—the L33P substitution in the β3 integrin gene—directly blocks ANDV entry [82]. These genetic discoveries have direct clinical and therapeutic implications. Screening for high-risk HLA haplotypes could enable early intensive monitoring and triage of vulnerable patients, while the protective integrin variant validates β3 integrin as a compelling therapeutic target for novel entry inhibitors [82]. Understanding these genetic determinants supports personalized risk stratification and informs future immunomodulatory and antiviral strategies, though validation across diverse populations and hantavirus species remains a research priority [83].

## 5. Environmental and Ecological Drivers

### 5.1. Climate Change and Hantavirus Emergence

**Figure 5:**
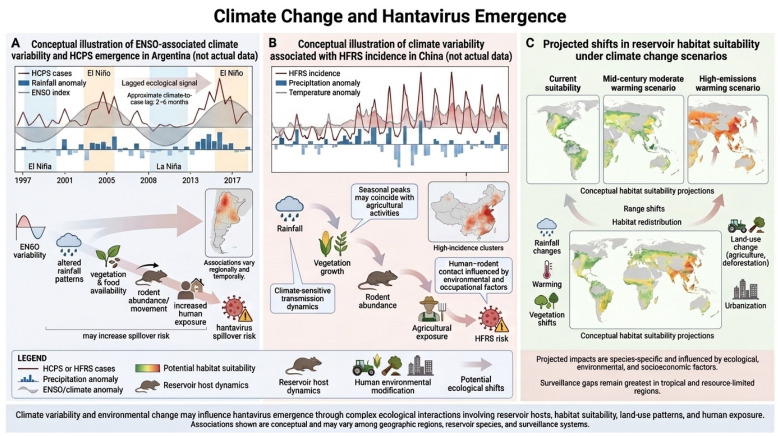
Climate change and hantavirus emergence. Conceptual overview of how climate variability, environmental change, and reservoir host ecology may influence hantavirus emergence within a One Health framework. Panels (**A**,**B**) are schematic illustrations synthesized from published observations and proposed ecological relationships and do not represent original or reconstructed epidemiological datasets. Panel (**A**) depicts the hypothesized influence of El Niño–Southern Oscillation (ENSO)-associated rainfall variability on environmental conditions, reservoir host dynamics, and hantavirus cardiopulmonary syndrome (HCPS) emergence in Argentina. Panel (**B**) illustrates conceptual pathways linking temperature and precipitation variability to reservoir host abundance, human exposure, and hemorrhagic fever with renal syndrome (HFRS) risk in China. Panel (**C**) presents conceptual projections of potential shifts in reservoir habitat suitability and geographic distribution under future climate change scenarios, including the effects of warming, altered precipitation patterns, land-use change, and urbanization. Associations shown are intended to illustrate plausible mechanisms reported in the literature and may vary across geographic regions, reservoir species, surveillance systems, and environmental contexts.

Climate change is increasingly recognized as a major driver of hantavirus emergence by influencing rodent population dynamics, geographic distribution, and transmission risk [10,13,14]. Temperature changes, precipitation patterns, and ENSO events have been associated with increased hantavirus incidence and may support the development of climate-based early warning systems [84,85]. Climate-related expansion of rodent reservoirs into new habitats may further increase the risk of hantavirus spread to previously unaffected regions [12]. The interactions between climate variability, reservoir ecology, and hantavirus emergence are illustrated in Figure 5.

### 5.2. Land Use Change and Agricultural Expansion

Anthropogenic land-use changes, such as deforestation, agricultural expansion, and urbanization, can alter rodent habitats and increase human exposure to hantaviruses [13,48,84]. Studies from Brazil and China have linked environmental modification and agricultural intensification to increased hantavirus transmission risk and shifts in disease epidemiology [85,86].

### 5.3. Urbanization and Peri-Urban Transmission

Urbanization represents an increasingly vital driver of hantavirus transmission, especially for Seoul virus, which is carried by the Norway rat (*Rattus norvegicus*), a highly adaptable urban rodent [27,87]. Studies have documented Seoul virus circulation in urban rat populations in multiple countries, with sporadic human cases reported [88,89]. Long-term studies of urban rodent populations have revealed persistent hantavirus circulation in protected urban areas, with seroprevalence rates varying seasonally in association with rodent population dynamics and weather patterns [90]. These findings highlight the need for urban rodent control measures and surveillance in cities where human-rat contact is frequent [91].

### 5.4. Biodiversity and Dilution Effects

The relationship between biodiversity and hantavirus risk is complex, as higher biodiversity may reduce transmission through dilution effects, whereas biodiversity loss may favor reservoir species and increase transmission risk [92]. Studies suggest that biodiversity, climate, and socioeconomic factors interact to influence hantavirus emergence and should be considered in prevention strategies [93].

## 6. Clinical Manifestations and Diagnosis

### 6.1. Clinical Presentation of HFRS

HFRS typically presents with sudden onset of fever, headache, back pain, abdominal pain, and gastrointestinal symptoms [41,94]. The disease progresses through characteristic phases: febrile (3–7 days), hypotensive (hours to 3 days), oliguric (3–7 days), diuretic (days to weeks), and convalescent (weeks to months) [95]. Laboratory findings include thrombocytopenia, proteinuria, hematuria, and elevated creatinine [94]. Imaging findings in HFRS commonly include renal enlargement, perirenal fluid accumulation, and retroperitoneal edema, with hemorrhagic manifestations observed in severe disease [96]. The severity of HFRS varies by viral species, with Hantaan and Dobrava-Belgrade viruses typically causing more severe disease than Puumala virus [97].

### 6.2. Clinical Presentation of HCPS

HCPS typically begins with a prodromal phase characterized by fever, myalgia, headache, and gastrointestinal symptoms lasting 3–6 days [98]. This is followed by rapid onset of respiratory distress, pulmonary edema, and cardiogenic shock [99]. Characteristic laboratory findings include thrombocytopenia, hemoconcentration, elevated lactate dehydrogenase, and immunoblastic lymphocytes [7]. Chest imaging reveals bilateral interstitial infiltrates and pleural effusions [7]. The rapid progression from initial symptoms to respiratory failure necessitates early recognition and intensive supportive care [99]. Survivors typically experience complete recovery, though convalescence may be prolonged [98]. The clinical differences between the two syndromes are shown in Table 3.

### 6.3. Diagnostic Methods

Early and accurate diagnosis of hantavirus infection is critical for appropriate clinical management and public health response [100,101]. Diagnostic approaches include serological methods, molecular detection, and immunohistochemistry [16,102].

#### 6.3.1. Serological Diagnostics

Serological testing is the primary diagnostic approach for hantavirus infections, with enzyme-linked immunosorbent assay (ELISA)-based detection of IgM and IgG antibodies commonly used for initial screening [16]. Confirmatory methods, namely immunofluorescence assays (IFA), Western blot, and microneutralization tests, can improve specificity and allow species-level identification; however, cross-reactivity among hantavirus species may complicate interpretation in regions where multiple strains co-circulate [102].

#### 6.3.2. Molecular Diagnostics

Reverse transcription polymerase chain reaction (RT-PCR) enables direct detection of hantavirus RNA and is extremely useful for early diagnosis before antibody seroconversion [100]. Real-time RT-PCR assays targeting conserved viral regions provide rapid and highly sensitive detection, although their utility is limited by the rapid decline in viral RNA after symptom onset [101].

#### 6.3.3. Point-of-Care and Field-Deployable Diagnostics

**Figure 6:**
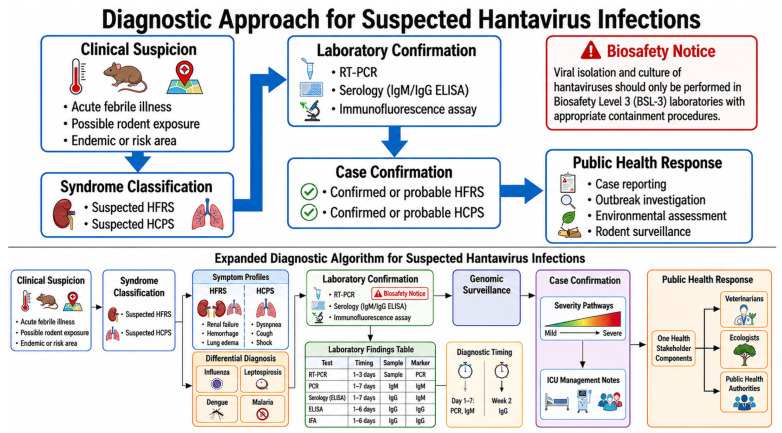
Diagnostic approach for suspected hantavirus infections. The upper panel presents a simplified diagnostic workflow for patients with suspected hantavirus infection based on epidemiologic exposure, clinical suspicion, laboratory confirmation, case classification, and public health response. The algorithm differentiates between hemorrhagic fever with renal syndrome (HFRS) and hantavirus cardiopulmonary syndrome (HCPS) and highlights the central role of molecular and serologic testing in diagnosis. A biosafety notice emphasizes that hantavirus isolation and viral culture require Biosafety Level 3 (BSL-3) containment. The lower panel provides additional diagnostic considerations, including syndrome-specific clinical features, differential diagnoses, laboratory findings, diagnostic timing, genomic surveillance, severity assessment, and One Health stakeholder involvement relevant to case investigation and outbreak response.

A proposed diagnostic workflow for suspected hantavirus infection is presented in Figure 6, and laboratory approaches for hantavirus detection are detailed in Table 4. Point-of-care diagnostics are increasingly crucial for hantavirus detection in resource-limited and outbreak settings [103]. Emerging approaches, comprising RT-LAMP and lateral flow immunoassays, offer rapid and field-deployable testing, although challenges related to sensitivity and specificity remain [104]. Ideal assays should provide rapid, accurate, and affordable detection with minimal equipment requirements [105].

### 6.4. Diagnostic Challenges in Tropical and Caribbean Settings

Diagnostic capacity for hantavirus infections in tropical and Caribbean regions faces multiple challenges [106]. Limited availability of validated serological assays, lack of molecular diagnostic infrastructure, absence of reference laboratories, and limited awareness among healthcare providers all contribute to underdiagnosis [107]. Additionally, the potential circulation of uncharacterized hantavirus species in these regions may limit the performance of existing diagnostic assays developed for well-characterized species [45]. Strengthening laboratory infrastructure and improving clinician awareness are essential for enhancing early detection and surveillance capacity in these settings [106]. Differential diagnoses of hantavirus infection in tropical settings are listed in Table 5.

## 7. Surveillance and Early Warning Systems

### 7.1. Current Surveillance Approaches

Hantavirus surveillance systems vary widely in scope and sophistication across different regions [108]. In countries with high disease burden, such as China and the Republic of Korea, national surveillance systems capture case data, conduct seroprevalence surveys, and monitor rodent populations [94,95]. These systems have generated valuable long-term datasets that have informed understanding of disease epidemiology and risk factors.

In contrast, many tropical and Caribbean countries lack systematic hantavirus surveillance, relying instead on passive case detection and sporadic research studies [15]. This surveillance gap limits understanding of disease burden, circulating viral species, and risk factors in these regions. In the case of the Italian traveler to Cuba, the infection was detected, diagnosed, and treated promptly due to the returning traveler’s access to advanced diagnostic infrastructure and clinicians’ awareness of imported zoonotic diseases—advantages that remain scarce in many local Caribbean settings [61].

### 7.2. Integrated One Health Surveillance

**Figure 7:**
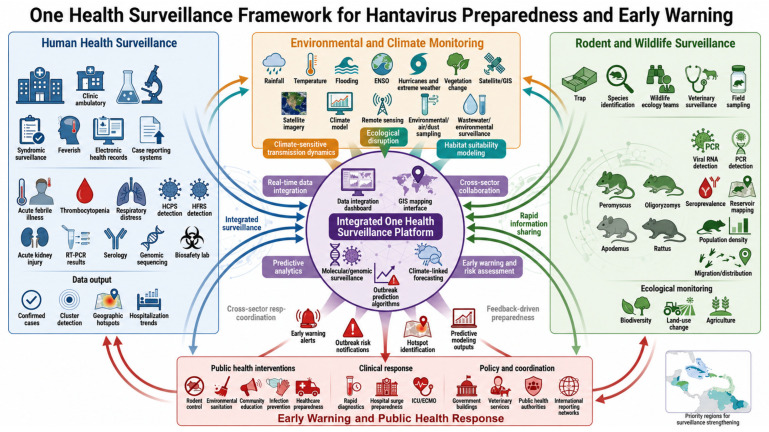
One Health surveillance framework for hantavirus preparedness and early warning. The framework illustrates the integration of human, animal, environmental, and public health sectors to support hantavirus surveillance, risk assessment, and outbreak preparedness. The human health surveillance component includes clinical case detection, laboratory diagnostics, syndromic surveillance, electronic health records, and genomic surveillance used to identify and monitor human infections. The environmental and climate monitoring sector incorporates climatic, ecological, and geospatial factors, including rainfall, temperature, flooding, ENSO activity, land-use change, remote sensing, and environmental sampling, that influence reservoir ecology and transmission risk. The rodent and wildlife surveillance sector focuses on reservoir host monitoring, species identification, population dynamics, pathogen detection, sero-surveillance, and ecological assessment. These data streams converge within an integrated One Health surveillance platform, where information is combined for real-time data integration, outbreak prediction, climate-linked forecasting, hotspot identification, and early warning generation. Outputs from the platform inform public health preparedness and response, including clinical management, infection prevention, environmental interventions, risk communication, policy development, and intersectoral coordination. The inset map highlights geographic regions where enhanced surveillance capacity, ecological monitoring, and preparedness efforts may be particularly valuable because of current surveillance gaps, environmental risk factors, or emerging hantavirus activity.

The One Health approach integrates human, animal, and environmental surveillance systems to improve hantavirus monitoring, risk assessment, and outbreak preparedness [20]. Human surveillance focuses on clinical case detection and laboratory confirmation, while rodent surveillance evaluates reservoir distribution, population dynamics, and viral prevalence [30]. Environmental surveillance incorporates climate variability, land-use change, and ecological factors associated with transmission risk [47]. The integration of these surveillance streams improves early detection of changing transmission patterns and supports targeted public health interventions. The interconnected surveillance framework and data flows are illustrated in Figure 7.

### 7.3. Climate-Based Early Warning Systems

Associations between climate variables and hantavirus incidence have prompted the development of climate-based early warning systems that integrate environmental data, rodent surveillance, and predictive models to identify periods of increased transmission risk [10,12]. Studies in China and the Americas suggest that temperature, precipitation, and El Niño patterns may help forecast outbreaks and support public health preparedness [14].

### 7.4. Surveillance Priorities for Tropical and Caribbean Regions

**Figure 8:**
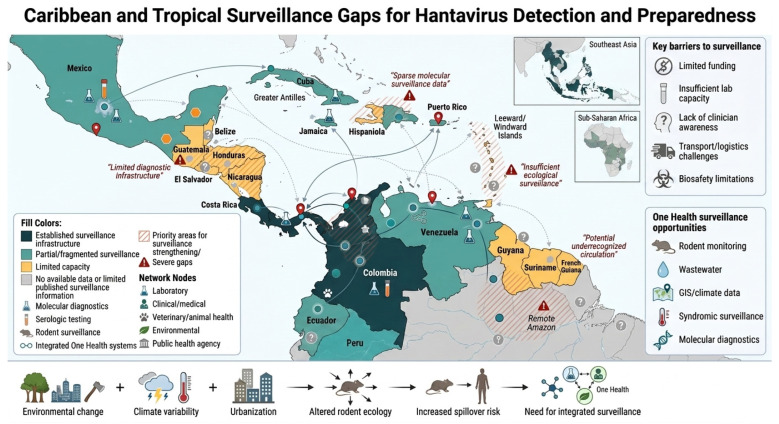
Surveillance gaps and preparedness priorities for hantavirus detection in Caribbean and tropical regions. Regional map illustrating variations in hantavirus surveillance capacity across the Caribbean basin and selected tropical regions of Central and South America. Areas with documented hantavirus activity and regions with limited surveillance infrastructure are highlighted to emphasize the potential for underrecognized transmission. The figure identifies priority areas for strengthening laboratory capacity, rodent and wildlife surveillance, clinician awareness, molecular diagnostics, and environmental monitoring. Map colors represent relative surveillance capacity, infrastructure, and One Health integration rather than the absolute presence or absence of surveillance programs. Countries classified as having limited or fragmented surveillance may still maintain national reporting systems, diagnostic capabilities, or localized hantavirus investigations. Priority regions highlighted on the map represent areas where enhanced surveillance, ecological monitoring, laboratory capacity, and preparedness efforts may improve early detection and outbreak response.

Strengthening hantavirus surveillance in tropical and Caribbean regions requires improved epidemiological research, diagnostic capacity, healthcare awareness, and regional collaboration [11,16]. Integrating hantavirus monitoring into existing infectious disease surveillance systems may provide a practical and cost-effective approach to improving preparedness [11]. Major surveillance gaps and preparedness priorities are summarized in Figure 8, while surveillance challenges and proposed solutions are outlined in Table 6.

## 8. Prevention and Control

### 8.1. Prevention of Human Exposure

Primary prevention of hantavirus focuses on reducing human exposure to infected rodent excreta through rodent-proofing, sanitation, safe cleanup practices, use of personal protective equipment, and public education [5,54]. Enhanced precautions are crucial in high-risk occupational and outdoor settings, including farming, forestry, construction, and military activities [45].

### 8.2. Rodent Control

Rodent control is an important strategy for reducing hantavirus transmission risk, although long-term effectiveness may be limited by ecological and logistical challenges [109]. Habitat modification, sanitation measures, trapping, and targeted rodent reduction programs are commonly used to reduce human exposure in high-risk settings [84]. The use of rodenticides may be effective in some contexts but raises concerns regarding environmental impact and non-target species exposure [110]. Integrated pest management (IPM) strategies that combine environmental sanitation, habitat reduction, surveillance, and selective rodent control are generally recommended for sustainable prevention efforts [111]. Urban rodent control is essential for preventing Seoul virus transmission associated with Norway rats in densely populated cities [91].

### 8.3. Vaccines

Currently, no hantavirus vaccine is licensed outside of China and the Republic of Korea, where inactivated vaccines targeting Hantaan and Seoul viruses provide only limited, short-lived immunity [99,112]. To address this gap, next-generation platforms—particularly mRNA and virus-like particle (VLP) vaccines—have shown promising preclinical results [113,114]. mRNA-LNP vaccines encoding Andes or Hantaan virus glycoproteins have elicited high-titer neutralizing antibodies and complete protection in rodent challenge models [115,116]. Similarly, VLPs displaying conformationally intact Gn/Gc spikes induce robust B- and T-cell responses superior to inactivated vaccines [117,118]. These platforms also enable rapid, scalable production of multivalent formulations capable of targeting multiple hantavirus serotypes simultaneously, though none have yet advanced beyond preclinical stages.

Concurrent advances in neutralizing antibody characterization have identified key correlates of protection. High-resolution in situ cryo-electron microscopy studies of ANDV, a highly pathogenic hantavirus, have resolved the architecture of the Gn/Gc glycoprotein tetramer and its organization on the virion surface [25]. These findings provide crucial insights into spike assembly, glycoprotein interactions, and antigen presentation [119]. Importantly, these structural models have enabled the identification of key neutralizing antibody binding sites and have refined our understanding of the molecular determinants of protective immunity [119]. Such knowledge facilitates structure-guided vaccine development by allowing the design of immunogens that closely mimic the native conformation of viral surface glycoproteins—an approach likely to enhance the induction of broadly neutralizing antibody responses [120]. Furthermore, these structural insights may aid in the development of multivalent vaccine candidates designed to elicit cross-protective immunity against diverse hantavirus species, including those responsible for both HFRS and HCPS [25]. While these findings open avenues for universal hantavirus vaccines, the present manuscript reviews published data rather than presenting new experimental results; accordingly, claims of rapid cross-protective efficacy should be interpreted as future potential, not current evidence.

### 8.4. Therapeutics

Presently, no FDA-approved antiviral treatments exist for hantavirus infection, and clinical management remains largely supportive. For HFRS, this includes fluid management and dialysis, while severe HCPS often requires intensive care, mechanical ventilation, or extracorporeal membrane oxygenation [121,122]. The broad-spectrum nucleoside analog ribavirin may reduce mortality in HFRS when administered early, although the supporting evidence is limited and no benefit has been demonstrated for HCPS [112]. Other repurposed agents, such as favipiravir, have shown in vitro activity but lack clinical data [123].

Emerging immunotherapies represent a more promising avenue, particularly neutralizing monoclonal antibodies (mAbs) targeting the viral Gn and Gc glycoproteins [124]. Preclinical studies, including in vivo models of ANDV infection, have demonstrated robust protective efficacy with certain mAbs, even when administered post-exposure [113,125]. Recent structural advances, including high-resolution cryo-electron microscopy of viral spike complexes, have identified conserved neutralizing epitopes that can guide the rational design of broadly reactive mAbs [25,115]. These findings support the continued development of mAb-based therapeutics as the most viable path toward a targeted, clinically approved treatment for hantavirus disease [126].

## 9. Research Priorities and Future Directions

### 9.1. Surveillance and Diagnostic Innovation

Advancing surveillance and diagnostic capabilities is essential for improving hantavirus detection and outbreak preparedness, especially in underrepresented tropical and Caribbean regions. Priority areas include the development of genomic surveillance platforms, rapid point-of-care diagnostics, digital surveillance systems, and standardized reporting frameworks [100,102]. Artificial intelligence, machine learning approaches, and environmental monitoring tools may further improve outbreak prediction and risk mapping by integrating climate, ecological, and epidemiological data [127]. Collectively, these innovations may strengthen early detection and public health response capacity [128].

### 9.2. Ecological and Environmental Research

Understanding hantavirus emergence requires integrated ecological, environmental, and epidemiological research. Key priorities include ecological niche modeling, longitudinal rodent surveillance, and studies evaluating the effects of climate change, biodiversity loss, land-use change, and environmental disruption on reservoir ecology and transmission risk [129]. Incorporating hantavirus risk assessments into environmental and land-use planning may help reduce future spillover risk [84].

### 9.3. Pathogenesis and Immunology

Improving understanding of hantavirus pathogenesis and host immune responses is important for the development of vaccines and therapeutics. Key research priorities include identifying mechanisms of endothelial dysfunction, vascular permeability, and immune-mediated injury associated with severe disease [65]. Studies investigating protective immunity, host genetic susceptibility, and improved animal models may further support the development of targeted therapies and vaccine strategies [78,83].

### 9.4. Vaccines and Therapeutics

Current research priorities for hantavirus vaccines and therapeutics focus on developing broadly protective and rapidly deployable countermeasures. Major areas of investigation include multivalent vaccines, mRNA vaccine platforms, monoclonal antibody therapies, and antiviral agents targeting viral replication or entry pathways [112,113]. Immunomodulatory therapies aimed at reducing excessive inflammatory responses may also improve outcomes in severe hantavirus disease [98,99].

### 9.5. Capacity Building in Underrepresented Regions

Addressing hantavirus surveillance and research gaps in tropical and Caribbean regions requires sustained investment in laboratory infrastructure, workforce training, and regional collaboration [106]. Priority efforts include strengthening diagnostic and genomic surveillance capacity, improving outbreak investigation expertise, and establishing collaborative regional networks for data sharing and coordinated response activities [107]. Targeted funding and South–South collaboration may further support sustainable preparedness and surveillance development in under-resourced regions [130,131].

## 10. One Health Framework for Hantavirus Preparedness

### 10.1. Integrating Human, Animal, and Environmental Health

The One Health framework recognizes the interconnected relationship between human, animal, and environmental health and is highly relevant to hantavirus preparedness [132]. Effective preparedness requires collaboration among public health agencies, veterinary services, environmental scientists, and wildlife experts to support integrated surveillance and coordinated outbreak response [133]. Shared surveillance systems that combine human case data, rodent ecology, and environmental monitoring may improve early detection of emerging transmission hotspots and support timely interventions.

### 10.2. Lessons from COVID-19 for Hantavirus Preparedness

The COVID-19 pandemic highlighted the importance of rapid surveillance, decentralized diagnostic capacity, international collaboration, and transparent public health communication for emerging infectious disease preparedness [134]. These lessons emphasize the need for integrated surveillance systems, rapid data sharing, and flexible public health infrastructure to strengthen hantavirus preparedness and outbreak response [18,134].

### 10.3. Building Resilient Health Systems

Strengthening health systems for emerging infectious disease preparedness requires sustained investment in surveillance, laboratory diagnostics, outbreak response capacity, and workforce development [25]. Flexible public health infrastructure and coordinated partnerships among governments, academic institutions, and international organizations are essential for effective preparedness and response [11]. Resilient and adaptable health systems will be critical for responding to future hantavirus outbreaks and other emerging zoonotic threats. One Health preparedness strategies are shown in Table 7.

## 11. Limitations and Methodological Challenges

Despite the synthesis presented in this review, our ability to accurately characterize hantavirus epidemiology in tropical and Caribbean regions remains constrained by several fundamental methodological challenges. Below, we address these critical challenges, as they highlight core limitations and define priorities for future research.

### 11.1. Estimating True Incidence

Important gaps remain in hantavirus surveillance, diagnostics, and disease recognition, most notably in tropical and resource-limited regions where infections may be underdiagnosed due to overlap with other endemic febrile illnesses and limited access to molecular and serological testing [22,23]. Accurately estimating hantavirus incidence, hospitalization, and mortality in tropical and Caribbean regions is challenging due to fragmented surveillance. Capture–recapture analysis using multiple incomplete data sources (hospital discharges, mortality registries, laboratory records) can estimate total cases missed by all systems. Systematic biases include diagnostic access bias (only severe cases tested, such as the case of the Italian traveler to Cuba, thus underestimating mild disease), clinical mimicry bias (misclassification as dengue or leptospirosis), and denominator uncertainty from transient populations [23]. Future studies should apply Bayesian hierarchical models that simultaneously correct for these biases, yielding adjusted incidence estimates likely to be different from those currently reported. In addition, variability in surveillance systems, diagnostic methods, and case definitions further limits comparisons across regions and may underestimate the global disease burden, highlighting the need for strengthened surveillance, standardized reporting, and integrated One Health approaches [20,21].

### 11.2. Quantifying Regional Differences in Climate Drivers

Comparing climate-driven hantavirus transmission across ecologically distinct regions—such as northern China (HFRS, temperate continental), Patagonia (HCPS, Andean Andes virus), and the Caribbean (tropical islands)—requires a standardized analytical framework. Distributed lag nonlinear models (DLNM) with identical climate variables (rainfall, temperature, ENSO, NDVI) and lag windows allow direct comparison of relative risks, while the proposed Climate Driver Intensity Index ranks driver relevance independent of regional baselines [12,15]. Applying this framework to published data from China, Argentina, and the Caribbean would reveal, for example, whether post-drought rainfall drives transmission in tropical islands as strongly as summer rainfall drives HFRS in northern China or ENSO drives HCPS in Patagonia [15,51,84]. Additionally, the ecological drivers of hantavirus emergence, including climate change, land-use change, and biodiversity loss, remain incompletely understood [12,13,14,48].

### 11.3. Low-Cost One Health Monitoring

A low-cost, replicable One Health system for Caribbean settings can link rodent monitoring to human case early warning without high-end equipment using simple rule-based algorithms. For example, monthly trapping with low cost Sherman traps (or homemade bottle traps) and filter paper blood spots, with samples sent in batches for RT-PCR at a central regional lab [135]. The critical linkage is achieved through a threshold trigger: when rodent abundance exceeds 20% above the 3-month moving average and recent rainfall is anomalous (>1 SD), an SMS alert is sent to district epidemiologists using free or low-cost gateways [136]. Clinic staff can be alerted through mobile health platforms, an approach successfully use in infectious disease surveillance in resource-limited settings and directly adaptable for hantaviruses [137].

### 11.4. Limited Therapy and Vaccines

Despite progress in vaccine and therapeutic research, no internationally licensed or globally available hantavirus vaccine currently exists, although inactivated vaccines against Hantaan and Seoul viruses are licensed and used in China and the Republic of Korea [99,113]. Furthermore, no specific antiviral therapy has demonstrated consistent clinical efficacy across hantavirus infections [112,113,114].

## 12. Conclusions

Hantaviruses remain critical emerging zoonotic pathogens with significant global health implications, primarily in regions where environmental change, urbanization, and climate variability are altering reservoir ecology and increasing opportunities for human exposure. The pathogenesis of hantavirus infections is driven largely by immune-mediated endothelial dysfunction and vascular permeability, leading to severe clinical syndromes including HFRS and HCPS. Despite advances in understanding hantavirus virology, immunopathology, diagnostics, and reservoir ecology, major gaps persist in surveillance infrastructure, diagnostic capacity, and epidemiological data. These gaps are especially evident in tropical and Caribbean regions, where hantavirus transmission may be underrecognized.

Recent progress in molecular diagnostics, genomic surveillance, vaccine platforms, monoclonal antibody therapies, and climate-informed risk modeling provides vital opportunities to improve early detection, preparedness, and outbreak response. However, sustained investment in laboratory infrastructure, ecological surveillance, workforce training, and regional collaboration remains essential. Integrated One Health approaches that combine human, animal, and environmental surveillance will be critical for strengthening global preparedness and reducing the impact of future hantavirus outbreaks and other emerging zoonotic threats.

## Figures and Tables

**Table 1:** Major hantaviruses, reservoirs, clinical syndrome, and geographic distribution.

Virus	Lineage	Primary Reservoir	Syndrome	Geographic Distribution
Hantaan (HTNV)	Old World	*Apodemus agrarius*	HFRS (severe)	China, Korea, Russia
Seoul (SEOV)	Old World	*Rattus norvegicus*	HFRS (mild)	Global (urban)
Puumala (PUUV)	Old World	*Myodes glareolus*	NE/HFRS (mild)	Scandinavia, W. Europe
Dobrava-Belgrade (DOBV)	Old World	*Apodemus flavicollis*	HFRS (severe)	Balkans, C. Europe
Sin Nombre (SNV)	New World	*Peromyscus maniculatus*	HCPS	North America
Andes (ANDV)	New World	*Oligoryzomys longicaudatus*	HCPS	South America
Laguna Negra (LNV)	New World	*Calomys laucha*	HCPS	Paraguay, Bolivia
Choclo	New World	*Oligoryzomys fulvescens*	HCPS	Panama, Central America

Note: Adapted from [2,5,24,30,41].

**Table 2:** Seroprevalence of hantaviruses in the Caribbean.

Country	Sample	Seroprevalence (IgG)	RT-PCR	Year(s)
Barbados [58]	Febrile patients	15.3%	Not performed	2008–2016
Barbados [59]	Wild rodents	3.8%	Negative	2019
Colombia (Caribbean region) [62]	Health center patients	1.0%	Not performed	2012–2013
Cuba [61]	Italian traveler (single case)	100.0%	Positive	2010
Suriname [22]	Patients suspected of leptospirosis	0.0%	Not performed	2008–2012, 2014
Trinidad [60]	Farm, abattoir, and office workers	11.4%	Not performed	2006
Antigua, Belize, Bermuda, Dominica, Grenada, Jamaica, Montserrat, St. Kitts, St. Lucia, St. Vincent [23]	Healthy pregnant women	0.0%	Not performed	2009–2011

IgG—immunoglobulin G; RT-PCR—reverse transcriptase polymerase chain reaction.

**Table 3:** Clinical differences between HFRS and HCPS.

Feature	HFRS	HCPS
Primary organ	Kidney	Lungs/Cardiovascular
Clinical phases	5 (febrile, hypotensive, oliguric, diuretic, convalescent)	2 (prodromal, cardiopulmonary)
Key manifestations	AKI, hemorrhage, hypotension	Pulmonary edema, respiratory failure
Thrombocytopenia	Yes	Yes (severe)
Mortality	0.5–15%	20–50%
Associated viruses	HTNV, PUUV, SEOV, DOBV	SNV, ANDV, Choclo, Laguna Negra
Geographic focus	Asia, Europe	Americas

Note: Adapted from [5,16,45,53,54,55,56,99].

**Table 4:** Laboratory diagnostic approaches for hantavirus detection.

Method	Specimen	Timing	Sensitivity	Specificity	BSL Required
IgM ELISA	Serum	Acute (≥3 days)	85–95%	90–95%	BSL-2
IgG ELISA	Serum	Acute/convalescent	90–98%	90–98%	BSL-2
RT-PCR	Blood, tissue	Early acute	70–90%	>95%	BSL-2 (post-extraction)
Immunohistochemistry	Tissue	Any (autopsy)	80–95%	>95%	BSL-2
Viral culture	Blood, tissue	Acute phase	Variable	100%	BSL-3
Rapid lateral flow	Whole blood	Acute phase	70–85%	85–95%	BSL-2

Note: Adapted from [16,100,101,102,103].

**Table 5:** Differential diagnosis of hantavirus infection in tropical settings.

Condition	Shared Features	Distinguishing Features	Key Test
Dengue fever	Fever, thrombocytopenia, myalgia	Rash, NS1 antigen, no renal failure	NS1 ELISA, RT-PCR
Leptospirosis	Fever, renal failure, myalgia	Jaundice, conjunctival suffusion	MAT, IgM ELISA
Malaria	Fever, thrombocytopenia, myalgia	Cyclic fever, splenomegaly	Blood smear, RDT
Influenza	Fever, myalgia, respiratory	No renal failure, no thrombocytopenia	Rapid influenza test
COVID-19	Fever, respiratory, myalgia	SARS-CoV-2 exposure, anosmia	RT-PCR
Rickettsial disease	Fever, headache, thrombocytopenia	Rash, eschar, tick exposure	Serology, PCR
Hantavirus	Fever, thrombocytopenia, renal/pulmonary	Rodent exposure, pulmonary edema, AKI	IgM ELISA, RT-PCR

Note: Adapted from [5,16,45].

**Table 6:** Surveillance challenges and proposed solutions in Caribbean and tropical regions.

Challenge	Impact	Proposed Solution
Limited molecular diagnostics	Missed cases, delayed outbreak detection	Regional RT-PCR platform sharing, training programs
No baseline seroprevalence data	Unknown disease burden	Population-based serosurveys
Clinical overlap with dengue/leptospirosis	Systematic misdiagnosis	Multiplex diagnostic panels, clinician education
No rodent surveillance	Undetected reservoir activity	Integrated rodent trapping and molecular testing
Fragmented reporting systems	Delayed outbreak recognition	Syndromic surveillance integration
Limited biosafety infrastructure	Inability to manage BSL-3 specimens	Biosafety capacity building, regional reference labs
Extreme weather events	Post-disaster exposure spikes	Disaster-response hantavirus surveillance protocols

Note: Authors synthesis based on [5,44,45].

**Table 7:** One Health preparedness strategies for hantavirus surveillance.

Sector	Key Activities	Expected Outcomes
Human health	Case surveillance, clinician training, syndromic systems	Improved case detection, reduced diagnostic delays
Veterinary/wildlife	Rodent trapping, viral testing, species mapping	Reservoir identification, early outbreak warning
Environmental	GIS mapping, climate modeling, environmental sampling	Risk area identification, predictive modeling
Laboratory	RT-PCR capacity, serology, biosafety, reference labs	Rapid confirmation, quality-assured diagnostics
Public health policy	Regional networks, data sharing, standardized protocols	Coordinated outbreak response, harmonized surveillance
Research	Seroprevalence studies, genomic surveillance, vaccine development	Evidence base, preparedness tools

Note: Authors framework adapted from [10,47,85,106,109].

## Data Availability

No new data was generated. All cited references are publicly available in PubMed or other indexed databases.

## Abbreviations

The following abbreviations are used in this manuscript:

ANDV	Andes virus
COVID-19	Coronavirus disease 2019
DOBV	Dobrava-Belgrade virus
ECMO	Extracorporeal membrane oxygenation
ELISA	Enzyme-linked immunosorbent assay
ENSO	El Niño Southern Oscillation
FDA	Food and Drug Administration
HCPS	Hantavirus cardiopulmonary syndrome
HFRS	Hemorrhagic fever with renal syndrome
HLA	Human leukocyte antigen
HTNV	Hantaan virus
IFA	Immunofluorescence assay
LAMP	Loop-mediated isothermal amplification
LNV	Laguna Negra virus
PUUV	Puumala virus
RT-PCR	Reverse transcription polymerase chain reaction
SEOV	Seoul virus
SNV	Sin Nombre virus

## References

[1] Wang S., Li W., Wang Z., Yang W., Li E., Xia X., Yan F., Chiu S. (2024). Emerging and Reemerging Infectious Diseases: Global Trends and New Strategies for Their Prevention and Control. Signal Transduct. Target. Ther..

[2] Taylor S.L., Schmaljohn C.S., Williams E.P., Jonsson C.B. (2025). Pathogenicity and Virulence of Rodent-Borne Orthohantaviruses. Virulence.

[3] Ksiazek T.G., Peters C.J., Rollin P.E., Zaki S., Nichol S., Spiropoulou C., Morzunov S., Feldmann H., Sanchez A., Khan A.S. (1995). Identification of a New North American Hantavirus That Causes Acute Pulmonary Insufficiency. Am. J. Trop. Med. Hyg..

[4] Korva M., Rus K.R., Pavletič M., Saksida A., Knap N., Jelovšek M., Smrdel K.S., Jakupi X., Humolli I., Dedushaj J. (2019). Characterization of Biomarker Levels in Crimean-Congo Hemorrhagic Fever and Hantavirus Fever with Renal Syndrome. Viruses.

[5] Jonsson C.B., Figueiredo L.T.M., Vapalahti O. (2010). A Global Perspective on Hantavirus Ecology, Epidemiology, and Disease. Clin. Microbiol. Rev..

[6] Armien B., Pascale J.M., Muñoz C., Mariñas J., Núñez H., Herrera M., Trujillo J., Sánchez D., Mendoza Y., Hjelle B. (2013). Hantavirus Fever without Pulmonary Syndrome in Panama. Am. J. Trop. Med. Hyg..

[7] Vial P.A., Ferrés M., Vial C., Klingström J., Ahlm C., López R., Le Corre N., Mertz G.J. (2023). Hantavirus in Humans: A Review of Clinical Aspects and Management. Lancet Infect. Dis..

[8] Martinez-Valdebenito C., Calvo M., Vial C., Mansilla R., Marco C., Palma R.E., Vial P.A., Valdivieso F., Mertz G., Ferrés M. (2014). Person-to-Person Household and Nosocomial Transmission of Andes Hantavirus, Southern Chile, 2011. Emerg. Infect. Dis..

[9] Guterres A., de Lemos E.R.S. (2018). Hantaviruses and a Neglected Environmental Determinant. One Health.

[10] Guo J., Semenza J.C., Ecke F., Rizzoli A., Dagostin F., Ulrich R.G., Sjödin H., Treskova M., Rocklöv J. (2026). A Pan-European Assessment of Multi-Sector Drivers of Human Hantavirus Risk: Climate, Biodiversity, and Socio-Economic Factors as Key Determinants. Environ. Res..

[11] Bhushan K., Kanwar S. (2020). The Negative Sense RNA Hantavirus—A Threat to the Modern World. Asian Pac. J. Health Sci..

[12] Prist P.R., Uriarte M., Fernandes K., Metzger J.P. (2017). Climate Change and Sugarcane Expansion Increase Hantavirus Infection Risk. PLoS Negl. Trop. Dis..

[13] Muylaert R.L., Sabino-Santos G.J., Prist P.R., Oshima J.E.F., Niebuhr B.B., Sobral-Souza T., de Oliveira S.V., Bovendorp R.S., Marshall J.C., Hayman D.T.S. (2019). Spatiotemporal Dynamics of Hantavirus Cardiopulmonary Syndrome Transmission Risk in Brazil. Viruses.

[14] Bai Y., Xu Z., Lu B., Sun Q., Tang W., Liu X., Yang W., Xu X., Liu Q. (2015). Effects of Climate and Rodent Factors on Hemorrhagic Fever with Renal Syndrome in Chongqing, China, 1997–2008. PLoS ONE.

[15] Douglas K.O., Payne K., Sabino-Santos G.J., Agard J. (2021). Influence of Climatic Factors on Human Hantavirus Infections in Latin America and the Caribbean: A Systematic Review. Pathogens.

[16] Mattar S., Guzmán C., Figueiredo L.T. (2015). Diagnosis of Hantavirus Infection in Humans. Expert Rev. Anti Infect. Ther..

[17] Sánchez-Lerma L., Mattar S., Contreras V., Miranda J., Tique V., Rodríguez V., Rodriguez D., Lopez S., Rojas-Gulloso A. (2025). Hantavirus and Leptospira Are Important Causes of Nonspecific Acute Febrile Syndrome, Meta, Colombia. Travel Med. Infect. Dis..

[18] Williams B.A., Jones C.H., Welch V., True J.M. (2023). Outlook of Pandemic Preparedness in a Post-COVID-19 World. npj Vaccines.

[19] Maccaro A., Audia C., Stokes K., Masud H., Sekalala S., Pecchia L., Piaggio D. (2023). Pandemic Preparedness: A Scoping Review of Best and Worst Practices from COVID-19. Healthcare.

[20] Desvars-Larrive A., Vogl A.E., Puspitarani G.A., Yang L., Joachim A., Käsbohrer A. (2024). A One Health Framework for Exploring Zoonotic Interactions Demonstrated through a Case Study. Nat. Commun..

[21] Berjaoui S., Puglia I., Caporale M., Pinoni C., Salvaggiulo A., Gatta G., Sepashvili M., Di Donato G. (2026). Hantaviruses in the One Health Era: Strengthening Surveillance before the next Spillover. Vet. Ital..

[22] Goeijenbier M., Aron G., Anfasa F., Lundkvist Å., Verner-Carlsson J., Reusken C.B.E.M., Martina B.E.E., van Gorp E.C.M., Resida L. (2015). Emerging Viruses in the Republic of Suriname: Retrospective and Prospective Study into Chikungunya Circulation and Suspicion of Human Hantavirus Infections, 2008–2012 and 2014. Vector Borne Zoonotic Dis..

[23] Wood H., Drebot M.A., Dewailly E., Dillon L., Dimitrova K., Forde M., Grolla A., Lee E., Loftis A., Makowski K. (2014). Seroprevalence of Seven Zoonotic Pathogens in Pregnant Women from the Caribbean. Am. J. Trop. Med. Hyg..

[24] Bradfute S.B., Calisher C.H., Klempa B., Klingström J., Kuhn J.H., Laenen L., Tischler N.D., Maes P. (2024). ICTV Virus Taxonomy Profile: Hantaviridae 2024. J. Gen. Virol..

[25] Guo L., McFadden E., Slough M.M., Stone E.T., Berrigan J., Mittler E., Hatzakis K., Hinkley T., Kain H.S., Ke Z. (2026). High-Resolution in Situ Structures of Hantavirus Glycoprotein Tetramers. Cell.

[26] Khaiboullina S.F., Morzunov S.P., St Jeor S.C. (2005). Hantaviruses: Molecular Biology, Evolution and Pathogenesis. Curr. Mol. Med..

[27] Lin X.-D., Guo W.-P., Wang W., Zou Y., Hao Z.-Y., Zhou D.-J., Dong X., Qu Y.-G., Li M.-H., Tian H.-F. (2012). Migration of Norway Rats Resulted in the Worldwide Distribution of Seoul Hantavirus Today. J. Virol..

[28] Brummer-Korvenkontio M., Vaheri A., Hovi T., von Bonsdorff C.H., Vuorimies J., Manni T., Penttinen K., Oker-Blom N., Lähdevirta J. (1980). Nephropathia Epidemica: Detection of Antigen in Bank Voles and Serologic Diagnosis of Human Infection. J. Infect. Dis..

[29] Klempa B., Schütt M., Auste B., Labuda M., Ulrich R., Meisel H., Krüger D.H. (2004). First Molecular Identification of Human Dobrava Virus Infection in Central Europe. J. Clin. Microbiol..

[30] Herbreteau V., Henttonen H., Yoshimatsu K., Gonzalez J.-P., Suputtamongkol Y., Hugot J.-P. (2006). Hantavirus Coevolution with Their Rodent Hosts. Encyclopedia of Infectious Diseases: Modern Methodologies.

[31] Nichol S.T., Spiropoulou C.F., Morzunov S., Rollin P.E., Ksiazek T.G., Feldmann H., Sanchez A., Childs J., Zaki S., Peters C.J. (1993). Genetic Identification of a Hantavirus Associated with an Outbreak of Acute Respiratory Illness. Science.

[32] López N., Padula P., Rossi C., Lázaro M.E., Franze-Fernández M.T. (1996). Genetic Identification of a New Hantavirus Causing Severe Pulmonary Syndrome in Argentina. Virology.

[33] Young J.C., Mills J.N., Enria D.A., Dolan N.E., Khan A.S., Ksiazek T.G. (1998). New World Hantaviruses. Br. Med. Bull..

[34] Wells R.M., Sosa Estani S., Yadon Z.E., Enria D., Padula P., Pini N., Mills J.N., Peters C.J., Segura E.L. (1997). An Unusual Hantavirus Outbreak in Southern Argentina: Person-to-Person Transmission? Hantavirus Pulmonary Syndrome Study Group for Patagonia. Emerg. Infect. Dis..

[35] Arai S., Kikuchi F., Bawm S., Sơn N.T., Lin K.S., Tú V.T., Aoki K., Tsuchiya K., Tanaka-Taya K., Morikawa S. (2019). Molecular Phylogeny of Mobatviruses (Hantaviridae) in Myanmar and Vietnam. Viruses.

[36] Laenen L., Vergote V., Kafetzopoulou L.E., Wawina T.B., Vassou D., Cook J.A., Hugot J.-P., Deboutte W., Kang H.J., Witkowski P.T. (2018). A Novel Hantavirus of the European Mole, Bruges Virus, Is Involved in Frequent Nova Virus Coinfections. Genome Biol. Evol..

[37] Guo W.-P., Lin X.-D., Wang W., Tian J.-H., Cong M.-L., Zhang H.-L., Wang M.-R., Zhou R.-H., Wang J.-B., Li M.-H. (2013). Phylogeny and Origins of Hantaviruses Harbored by Bats, Insectivores, and Rodents. PLoS Pathog..

[38] Klempa B. (2018). Reassortment Events in the Evolution of Hantaviruses. Virus Genes.

[39] Ramsden C., Holmes E.C., Charleston M.A. (2009). Hantavirus Evolution in Relation to Its Rodent and Insectivore Hosts: No Evidence for Codivergence. Mol. Biol. Evol..

[40] Easterbrook J.D., Klein S.L. (2008). Immunological Mechanisms Mediating Hantavirus Persistence in Rodent Reservoirs. PLoS Pathog..

[41] Tkachenko E., Dzagurova T., Galieva G., Ivanis V., Kurashova S., Tkachenko P., Balkina A., Trankvilevsky D., Ishmukhametov A. (2025). Clinical Manifestations of Hemorrhagic Fever with Renal Syndrome, Various Nosologic Forms and Issues of Hantavirus Infections Terminology. Viruses.

[42] Watson D.C., Sargianou M., Papa A., Chra P., Starakis I., Panos G. (2014). Epidemiology of Hantavirus Infections in Humans: A Comprehensive, Global Overview. Crit. Rev. Microbiol..

[43] Zhang Y.-Z., Zou Y., Fu Z.F., Plyusnin A. (2010). Hantavirus Infections in Humans and Animals, China. Emerg. Infect. Dis..

[44] Zhang S., Wang S., Yin W., Liang M., Li J., Zhang Q., Feng Z., Li D. (2014). Epidemic Characteristics of Hemorrhagic Fever with Renal Syndrome in China, 2006–2012. BMC Infect. Dis..

[45] Manigold T., Vial P. (2014). Human Hantavirus Infections: Epidemiology, Clinical Features, Pathogenesis and Immunology. Swiss Med. Wkly..

[46] Jiang H., Zheng X., Wang L., Du H., Wang P., Bai X. (2017). Hantavirus Infection: A Global Zoonotic Challenge. Virol. Sin..

[47] Tian H.-Y., Yu P.-B., Luis A.D., Bi P., Cazelles B., Laine M., Huang S.-Q., Ma C.-F., Zhou S., Wei J. (2015). Changes in Rodent Abundance and Weather Conditions Potentially Drive Hemorrhagic Fever with Renal Syndrome Outbreaks in Xi’an, China, 2005–2012. PLoS Negl. Trop. Dis..

[48] Tian H., Yu P., Bjørnstad O.N., Cazelles B., Yang J., Tan H., Huang S., Cui Y., Dong L., Ma C. (2017). Anthropogenically Driven Environmental Changes Shift the Ecological Dynamics of Hemorrhagic Fever with Renal Syndrome. PLoS Pathog..

[49] Koroknai A., Nagy A., Nagy O., Csonka N., Zsichla L., Szomor K., Takács M. (2026). Human Hantavirus Infections in Hungary (2018–2025): Epidemiology, Molecular Detection Across Clinical Sample Types, and Phylogenetic Analysis. Viruses.

[50] Tscherne A., Guardado-Calvo P., Clark J.J., Krause R., Krammer F. (2025). Puumala Orthohantavirus: Prevalence, Biology, Disease, Animal Models and Recent Advances in Therapeutics Development and Structural Biology. Front. Immunol..

[51] Sun L., Zou L.-X. (2018). Spatiotemporal Analysis and Forecasting Model of Hemorrhagic Fever with Renal Syndrome in Mainland China. Epidemiol. Infect..

[52] Whitmer S.L.M., Whitesell A., Mobley M., Talundzic E., Shedroff E., Cossaboom C.M., Messenger S., Deldari M., Bhatnagar J., Estetter L. (2024). Human Orthohantavirus Disease Prevalence and Genotype Distribution in the U.S., 2008–2020: A Retrospective Observational Study. Lancet Reg. Health—Am..

[53] Alonso D.O., Iglesias A., Coelho R., Periolo N., Bruno A., Córdoba M.T., Filomarino N., Quipildor M., Biondo E., Fortunato E. (2019). Epidemiological Description, Case-Fatality Rate, and Trends of Hantavirus Pulmonary Syndrome: 9 Years of Surveillance in Argentina. J. Med. Virol..

[54] Martínez Valeria P., Di Paola N., Alonso D.O., Pérez-Sautu U., Bellomo C.M., Iglesias A.A., Coelho R.M., López B., Periolo N., Larson P.A. (2020). “Super-Spreaders” and Person-to-Person Transmission of Andes Virus in Argentina. N. Engl. J. Med..

[55] Figueiredo L.T.M., Moreli M.L., de-Sousa R.L.M., Borges A.A., de-Figueiredo G.G., Machado A.M., Bisordi I., Nagasse-Sugahara T.K., Suzuki A., Pereira L.E. (2009). Hantavirus Pulmonary Syndrome, Central Plateau, Southeastern, and Southern Brazil. Emerg. Infect. Dis..

[56] Mendes W.S., da Silva A.A.M., Aragão L.F.C., Aragão N.J.L., de Raposo M.L., Elkhoury M.R., Suzuky A., Ferreira I.B., de Sousa L.T., Pannuti C.S. (2004). Hantavirus Infection in Anajatuba, Maranhao, Brazil. Emerg. Infect. Dis..

[57] Castel G., Filippone C., Tatard C., Vigan J., Dobigny G. (2023). Role of Seaports and Imported Rats in Seoul Hantavirus Circulation, Africa. Emerg. Infect. Dis..

[58] Douglas K.O., Samuels T.A., Iheozor-Ejiofor R., Vapalahti O., Sironen T., Gittens-St Hilaire M. (2021). Serological Evidence of Human Orthohantavirus Infections in Barbados, 2008 to 2016. Pathogens.

[59] Douglas K.O., Cayol C., Forbes K.M., Samuels T.A., Vapalahti O., Sironen T., Gittens-St Hilaire M. (2021). Serological Evidence of Multiple Zoonotic Viral Infections among Wild Rodents in Barbados. Pathogens.

[60] Adesiyun A., Dookeran S., Stewart-Johnson A., Rahaman S., Bissessar S., Thompson N. (2011). Serological Evidence of Hantavirus Infection in Farm and Abattoir Workers in Trinidad—A Preliminary Study. J. Agromed..

[61] Rovida F., Percivalle E., Sarasini A., Chichino G., Baldanti F. (2013). Imported Hantavirus Cardiopulmonary Syndrome in an Italian Traveller Returning from Cuba. New Microbiol..

[62] Bolaños A., Montoya-Ruiz C., Perez-Peréz J.C., Rodas J.D., Mattar S. (2019). Seroprevalence of Arenavirus and Hantavirus in Indigenous Populations from the Caribbean, Colombia. Rev. Soc. Bras. Med. Trop..

[63] Mittler E., Dieterle M.E., Kleinfelter L.M., Slough M.M., Chandran K., Jangra R.K. (2019). Hantavirus Entry: Perspectives and Recent Advances. Adv. Virus Res..

[64] Ermonval M., Baychelier F., Tordo N. (2016). What Do We Know about How Hantaviruses Interact with Their Different Hosts?. Viruses.

[65] Taylor S.L., Wahl-Jensen V., Copeland A.M., Jahrling P.B., Schmaljohn C.S. (2013). Endothelial Cell Permeability during Hantavirus Infection Involves Factor XII-Dependent Increased Activation of the Kallikrein-Kinin System. PLoS Pathog..

[66] Krautkrämer E., Grouls S., Hettwer D., Rafat N., Tönshoff B., Zeier M. (2014). Mobilization of Circulating Endothelial Progenitor Cells Correlates with the Clinical Course of Hantavirus Disease. J. Virol..

[67] Saavedra F., Díaz F.E., Retamal-Díaz A., Covián C., González P.A., Kalergis A.M. (2021). Immune Response during Hantavirus Diseases: Implications for Immunotherapies and Vaccine Design. Immunology.

[68] Klingström J., Smed-Sörensen A., Maleki K.T., Solà-Riera C., Ahlm C., Björkström N.K., Ljunggren H.G. (2019). Innate and Adaptive Immune Responses against Human Puumala Virus Infection: Immunopathogenesis and Suggestions for Novel Treatment Strategies for Severe Hantavirus-Associated Syndromes. J. Intern. Med..

[69] Rissanen I., Krumm S.A., Stass R., Whitaker A., Voss J.E., Bruce E.A., Rothenberger S., Kunz S., Burton D.R., Huiskonen J.T. (2021). Structural Basis for a Neutralizing Antibody Response Elicited by a Recombinant Hantaan Virus Gn Immunogen. mBio.

[70] Iglesias A.A., Períolo N., Bellomo C.M., Lewis L.C., Olivera C.P., Anselmo C.R., García M., Coelho R.M., Alonso D.O., Dighero-Kemp B. (2022). Delayed Viral Clearance despite High Number of Activated T Cells during the Acute Phase in Argentinean Patients with Hantavirus Pulmonary Syndrome. eBioMedicine.

[71] Kilpatrick E.D., Terajima M., Koster F.T., Catalina M.D., Cruz J., Ennis F.A. (2004). Role of Specific CD8+ T Cells in the Severity of a Fulminant Zoonotic Viral Hemorrhagic Fever, Hantavirus Pulmonary Syndrome. J. Immunol..

[72] Bui-Mansfield L.T., Cressler D.K. (2011). Imaging of Hemorrhagic Fever with Renal Syndrome: A Potential Bioterrorism Agent of Military Significance. Mil. Med..

[73] Bruno P., Hassell L.H., Brown J., Tanner W., Lau A. (1990). The Protean Manifestations of Hemorrhagic Fever with Renal Syndrome. A Retrospective Review of 26 Cases from Korea. Ann. Intern. Med..

[74] Wang R., Zhao X.-Y., Liu X.-J., Zhang M., Sun Y.-T., Ning X., Xu J., Bu C.-Y. (2023). Case Report: Multiple Organ Failure Caused by Hemorrhagic Fever with Renal Syndrome. Am. J. Trop. Med. Hyg..

[75] Llah S.T., Mir S., Sharif S., Khan S., Mir M.A. (2018). Hantavirus Induced Cardiopulmonary Syndrome: A Public Health Concern. J. Med. Virol..

[76] Chang B., Crowley M., Campen M., Koster F. (2007). Hantavirus Cardiopulmonary Syndrome. Semin. Respir. Crit. Care Med..

[77] Bondu V., Schrader R., Gawinowicz M.A., McGuire P., Lawrence D.A., Hjelle B., Buranda T. (2015). Elevated Cytokines, Thrombin and PAI-1 in Severe HCPS Patients Due to Sin Nombre Virus. Viruses.

[78] Ma Y., Yuan B., Yi J., Zhuang R., Wang J., Zhang Y., Xu Z., Zhang Y., Liu B., Wei C. (2012). The Genetic Polymorphisms of HLA Are Strongly Correlated with the Disease Severity after Hantaan Virus Infection in the Chinese Han Population. Clin. Dev. Immunol..

[79] Mustonen J., Partanen J., Kanerva M., Pietilä K., Vapalahti O., Pasternack A., Vaheri A. (1998). Association of HLA B27 with Benign Clinical Course of Nephropathia Epidemica Caused by Puumala Hantavirus. Scand. J. Immunol..

[80] Mäkelä S., Mustonen J., Ala-Houhala I., Hurme M., Partanen J., Vapalahti O., Vaheri A., Pasternack A. (2002). Human Leukocyte Antigen-B8-DR3 Is a More Important Risk Factor for Severe Puumala Hantavirus Infection than the Tumor Necrosis Factor-Alpha(-308) G/A Polymorphism. J. Infect. Dis..

[81] Wang M.L., Lai J.H., Zhu Y., Zhang H.B., Li C., Wang J.P., Li Y.M., Yang A.G., Jin B.Q. (2009). Genetic Susceptibility to Haemorrhagic Fever with Renal Syndrome Caused by Hantaan Virus in Chinese Han Population. Int. J. Immunogenet..

[82] Martínez-Valdebenito C., Angulo J., Le Corre N., Marco C., Vial C., Miquel J.F., Cerda J., Mertz G., Vial P., Lopez-Lastra M. (2019). A Single-Nucleotide Polymorphism of α(V)Β_3_ Integrin Is Associated with the Andes Virus Infection Susceptibility. Viruses.

[83] Saxenhofer M., Labutin A., White T.A., Heckel G. (2022). Host Genetic Factors Associated with the Range Limit of a European Hantavirus. Mol. Ecol..

[84] Muschetto E., Cueto G.R., Cavia R., Padula P.J., Suárez O.V. (2018). Long-Term Study of a Hantavirus Reservoir Population in an Urban Protected Area, Argentina. EcoHealth.

[85] Xiao H., Tian H.-Y., Gao L.-D., Liu H.-N., Duan L.-S., Basta N., Cazelles B., Li X.-J., Lin X.-L., Wu H.-W. (2014). Animal Reservoir, Natural and Socioeconomic Variations and the Transmission of Hemorrhagic Fever with Renal Syndrome in Chenzhou, China, 2006–2010. PLoS Negl. Trop. Dis..

[86] Pinto Junior V.L., Hamidad A.M., de Oliveira Albuquerque Filho D., dos Santos V.M. (2014). Twenty Years of Hantavirus Pulmonary Syndrome in Brazil: A Review of Epidemiological and Clinical Aspects. J. Infect. Dev. Ctries..

[87] McElhinney L.M., Marston D.A., Pounder K.C., Goharriz H., Wise E.L., Verner-Carlsson J., Jennings D., Johnson N., Civello A., Nunez A. (2017). High Prevalence of Seoul Hantavirus in a Breeding Colony of Pet Rats. Epidemiol. Infect..

[88] Knust B., Brown S., de St. Maurice A., Whitmer S., Koske S.E., Ervin E., Patel K., Graziano J., Morales-Betoulle M.E., House J. (2020). Seoul Virus Infection and Spread in United States Home-Based Ratteries: Rat and Human Testing Results from a Multistate Outbreak Investigation. J. Infect. Dis..

[89] Combs M., Byers K.A., Ghersi B.M., Blum M.J., Caccone A., Costa F., Himsworth C.G., Richardson J.L., Munshi-South J. (2018). Urban Rat Races: Spatial Population Genomics of Brown Rats (Rattus Norvegicus) Compared across Multiple Cities. Proc. Biol. Sci..

[90] Himsworth C.G., Parsons K.L., Jardine C., Patrick D.M. (2013). Rats, Cities, People, and Pathogens: A Systematic Review and Narrative Synthesis of Literature Regarding the Ecology of Rat-Associated Zoonoses in Urban Centers. Vector Borne Zoonotic Dis..

[91] Alburkat H., Smura T., Bouilloud M., Pradel J., Anfray G., Berthier K., Dutra L., Loiseau A., Niamsap T., Olander V. (2024). Evolution and Genetic Characterization of Seoul Virus in Wild Rats Rattus Norvegicus from an Urban Park in Lyon, France 2020–2022. PLoS Negl. Trop. Dis..

[92] Suzán G., Marcé E., Giermakowski J.T., Mills J.N., Ceballos G., Ostfeld R.S., Armién B., Pascale J.M., Yates T.L. (2009). Experimental Evidence for Reduced Rodent Diversity Causing Increased Hantavirus Prevalence. PLoS ONE.

[93] Keesing F., Holt R.D., Ostfeld R.S. (2006). Effects of Species Diversity on Disease Risk. Ecol. Lett..

[94] Park M.E., Kim D.Y., Seo J.-W., Yun N.R., Lee Y.M., Kim C.M., Kim D.-M. (2025). Analysis of Clinical and Laboratory Profiles of Patients Hospitalized with Hemorrhagic Fever with Renal Syndrome in Southwestern South Korea. Am. J. Trop. Med. Hyg..

[95] Zou L.-X., Chen M.-J., Sun L. (2016). Haemorrhagic Fever with Renal Syndrome: Literature Review and Distribution Analysis in China. Int. J. Infect. Dis..

[96] Paakkala A., Dastidar P., Ryymin P., Huhtala H., Mustonen J. (2005). Renal MRI Findings and Their Clinical Associations in Nephropathia Epidemica: Analysis of Quantitative Findings. Eur. Radiol..

[97] Kraft L., Latus J. (2026). Hantavirus Infections—Clinical Recognition and Diagnostic Challenges. Lancet Reg. Health—Eur..

[98] Singh P., Talwar P., Palaniyandi R. (2014). Hantavirus Pulmonary Syndrome (HPS): A Concise Review Based on Current Knowledge and Emerging Concept. J. Appl. Pharm. Sci..

[99] Brocato R.L., Hooper J.W. (2019). Progress on the Prevention and Treatment of Hantavirus Disease. Viruses.

[100] Geeraedts F., Wevers M., Bosma F., de Boer M., Brinkman J.N., Delsing C., GeurtsvanKessel C., Rockx B., van der Zanden A., Laverman G.D. (2024). Use of a Diagnostic Puumala Virus Real-Time RT-PCR in an Orthohantavirus Endemic Region in the Netherlands. Microbiol. Spectr..

[101] Niskanen S., Jääskeläinen A., Vapalahti O., Sironen T. (2019). Evaluation of Real-Time RT-PCR for Diagnostic Use in Detection of Puumala Virus. Viruses.

[102] Hoornweg T.E., Zutt I., De Vries A., Maas M., Hoogerwerf M.N., Avšič-Županc T., Korva M., Reimerink J.H.J., Reusken C.B.E.M. (2020). Development of a Comparative European Orthohantavirus Microneutralization Assay with Multi- Species Validation and Evaluation in a Human Diagnostic Cohort. Front. Cell. Infect. Microbiol..

[103] Zeng Y., Feng Y., Zhao Y., Zhang X., Yang L., Wang J., Gao Z., Zhang C. (2022). An HFman Probe-Based Multiplex Reverse Transcription Loop-Mediated Isothermal Amplification Assay for Simultaneous Detection of Hantaan and Seoul Viruses. Diagnostics.

[104] Huang C., Wei Q., Hu Q., Wen T., Xue L., Li S., Zeng X., Shi F., Jiao Y., Zhou L. (2019). Rapid Detection of Severe Fever with Thrombocytopenia Syndrome Virus (SFTSV) Total Antibodies by up-Converting Phosphor Technology-Based Lateral-Flow Assay. Luminescence.

[105] Adedokun G., Alipanah M., Fan Z.H. (2024). Sample Preparation and Detection Methods in Point-of-Care Devices towards Future at-Home Testing. Lab. Chip.

[106] Rodríguez Á., Couto P., Acevedo A., Herrera B.A., Astudillo O., Avaro M., Badillo G.B., Bruno A., Bustos P., Cerpa M. (2025). Strengthening the Surveillance and Response to Public Health Events with a One Health Approach: A Perspective from 12 Countries in Latin America and the Caribbean. J. Infect. Dis..

[107] Writer J.V., Kelley P.W., Boisson E.V., Hospedales J. (2003). Caribbean Public Health Laboratory Surveillance Project: A Department of Defense-Sponsored Humanitarian Mission. Mil. Med..

[108] Xu B., Yin Q., Ren D., Mo S., Ni T., Fu S., Zhang Z., Yan T., Zhao Y., Liu J. (2025). Scientometric Analysis of Research Trends in Hemorrhagic Fever with Renal Syndrome: A Historical Review and Network Visualization. J. Infect. Public Health.

[109] Tian H., Stenseth N.C. (2019). The Ecological Dynamics of Hantavirus Diseases: From Environmental Variability to Disease Prevention Largely Based on Data from China. PLoS Negl. Trop. Dis..

[110] Tosh D.G., Shore R.F., Jess S., Withers A., Bearhop S., Ian Montgomery W., McDonald R.A. (2011). User Behaviour, Best Practice and the Risks of Non-Target Exposure Associated with Anticoagulant Rodenticide Use. J. Environ. Manag..

[111] Brown L.M., Laco J. (2015). Rodent Control and Public Health: A Description of Local Rodent Control Programs. J. Environ. Health.

[112] Liu R., Ma H., Shu J., Zhang Q., Han M., Liu Z., Jin X., Zhang F., Wu X. (2019). Vaccines and Therapeutics Against Hantaviruses. Front. Microbiol..

[113] Hooper J.W., Brocato R.L., Kwilas S.A., Hammerbeck C.D., Josleyn M.D., Royals M., Ballantyne J., Wu H., Jiao J., Matsushita H. (2014). DNA Vaccine-Derived Human IgG Produced in Transchromosomal Bovines Protect in Lethal Models of Hantavirus Pulmonary Syndrome. Sci. Transl. Med..

[114] Qamar M.T.U., Ahmad S., Khan A., Wei D. (2024). Editorial: Immunotherapeutics Development against Hantaviruses. Front. Immunol..

[115] Aram M., Graham V., Kennedy E., Rayner E., Hewson R., Dowall S. (2025). A Multi-Valent Hantavirus Vaccine Based on Recombinant Modified Vaccinia Ankara Reduces Viral Load in a Mouse Infection Model. Vaccines.

[116] Ye W., Dang Y., Wang Y., Yang Q., Zhang H., Ye C., Wei J., Pei J., Pei X., Jiang D. (2026). Prefusion-Stabilized Hantaan Virus Glycoprotein Nucleic Acid Vaccine Elicits Potent Neutralizing Antibody Responses via Germinal Center Activation. Nat. Commun..

[117] Acuña R., Cifuentes-Muñoz N., Márquez C.L., Bulling M., Klingström J., Mancini R., Lozach P.-Y., Tischler N.D. (2014). Hantavirus Gn and Gc Glycoproteins Self-Assemble into Virus-like Particles. J. Virol..

[118] Zepeda-Cervantes J., Ramírez-Jarquín J.O., Vaca L. (2020). Interaction Between Virus-Like Particles (VLPs) and Pattern Recognition Receptors (PRRs) From Dendritic Cells (DCs): Toward Better Engineering of VLPs. Front. Immunol..

[119] Hooper J.W., Kwilas S.A., Josleyn M., Norris S., Hutter J.N., Hamer M., Livezey J., Paolino K., Twomey P., Koren M. (2024). Phase 1 Clinical Trial of Hantaan and Puumala Virus DNA Vaccines Delivered by Needle-Free Injection. npj Vaccines.

[120] Chai S., Wang L., Du H., Jiang H. (2025). Achievement and Challenges in Orthohantavirus Vaccines. Vaccines.

[121] Jiang H., Huang C., Bai X., Zhang F., Lin B., Wang S., Jia Z., Wang J., Liu J., Dang S. (2022). Expert Consensus on the Prevention and Treatment of Hemorrhagic Fever with Renal Syndrome. Infect. Dis. Immun..

[122] Wernly J.A., Dietl C.A., Tabe C.E., Pett S.B., Crandall C., Milligan K., Crowley M.R. (2011). Extracorporeal Membrane Oxygenation Support Improves Survival of Patients with Hantavirus Cardiopulmonary Syndrome Refractory to Medical Treatment. Eur. J. Cardiothorac. Surg..

[123] Mayor J., Engler O., Rothenberger S. (2021). Antiviral Efficacy of Ribavirin and Favipiravir against Hantaan Virus. Microorganisms.

[124] Duehr J., McMahon M., Williamson B., Amanat F., Durbin A., Hawman D.W., Noack D., Uhl S., Tan G.S., Feldmann H. (2020). Neutralizing Monoclonal Antibodies against the Gn and the Gc of the Andes Virus Glycoprotein Spike Complex Protect from Virus Challenge in a Preclinical Hamster Model. mBio.

[125] Engdahl T.B., Binshtein E., Brocato R.L., Kuzmina N.A., Principe L.M., Kwilas S.A., Kim R.K., Chapman N.S., Porter M.S., Guardado-Calvo P. (2023). Antigenic Mapping and Functional Characterization of Human New World Hantavirus Neutralizing Antibodies. eLife.

[126] Clark J.J., Hatzl S., Vasilev K., Andreata-Santos R., Yong J.S., Mittler E., Kasikci E., Chandran K., Simon V., Krause R. (2026). Cross-Binding Antibodies Capable of Neutralising Diverse Hantaviruses Are Produced in Response to Puumala Virus Infection. eBioMedicine.

[127] Zhang T., Rabhi F., Chen X., Paik H., MacIntyre C.R. (2024). A Machine Learning-Based Universal Outbreak Risk Prediction Tool. Comput. Biol. Med..

[128] Worsley-Tonks K.E.L., Bender J.B., Deem S.L., Ferguson A.W., Fèvre E.M., Martins D.J., Muloi D.M., Murray S., Mutinda M., Ogada D. (2022). Strengthening Global Health Security by Improving Disease Surveillance in Remote Rural Areas of Low-Income and Middle-Income Countries. Lancet Glob. Health.

[129] Danforth M.E., Messenger S., Buttke D., Weinburke M., Carroll G., Hacker G., Niemela M., Andrews E.S., Jackson B.T., Kramer V. (2020). Long-Term Rodent Surveillance after Outbreak of Hantavirus Infection, Yosemite National Park, California, USA, 2012. Emerg. Infect. Dis..

[130] Gongora V., Trotman M., Thomas R., Max M., Zamora P.A., Lepoureau M.T.F., Phanord S., Quirico J., Douglas K., Pegram R. (2008). The Caribbean Animal Health Network: New Tools for Harmonization and Reinforcement of Animal Disease Surveillance. Ann. N. Y. Acad. Sci..

[131] Belay E.D., Kile J.C., Hall A.J., Barton-Behravesh C., Parsons M.B., Salyer S., Walke H. (2017). Zoonotic Disease Programs for Enhancing Global Health Security. Emerg. Infect. Dis..

[132] Kelly T.R., Karesh W.B., Johnson C.K., Gilardi K.V.K., Anthony S.J., Goldstein T., Olson S.H., Machalaba C., Mazet J.A.K. (2017). One Health Proof of Concept: Bringing a Transdisciplinary Approach to Surveillance for Zoonotic Viruses at the Human-Wild Animal Interface. Prev. Vet. Med..

[133] Buregyeya E., Atusingwize E., Nsamba P., Musoke D., Naigaga I., Kabasa J.D., Amuguni H., Bazeyo W. (2020). Operationalizing the One Health Approach in Uganda: Challenges and Opportunities. J. Epidemiol. Glob. Health.

[134] Lau D.T., Sosa P., Dasgupta N., He H. (2021). Impact of the COVID-19 Pandemic on Public Health Surveillance and Survey Data Collections in the United States. Am. J. Public Health.

[135] Jakoniko J.R., Massawe A., Mwega E.D., Kessy S.T. (2024). Flea Burden on Rodents and Its Associated Determinants in Plague-Endemic Localities of Karatu District, Tanzania: A Cross-Sectional Study. Public Health Chall..

[136] He J., Christakos G., Zhang W., Wang Y. (2017). A Space-Time Study of Hemorrhagic Fever with Renal Syndrome (HFRS) and Its Climatic Associations in Heilongjiang Province, China. Front. Appl. Math. Stat..

[137] Mustafa U.-K., Kreppel K.S., Brinkel J., Sauli E. (2023). Digital Technologies to Enhance Infectious Disease Surveillance in Tanzania: A Scoping Review. Healthcare.

